# Lipodystrophy among HIV-Infected Patients Attending Care and Treatment Clinics in Dar es Salaam

**DOI:** 10.1155/2017/3896539

**Published:** 2017-10-11

**Authors:** Marina Njelekela, Rose Mpembeni, Alfa Muhihi, Nzovu Ulenga, Eric Aris, Deodatus Kakoko

**Affiliations:** ^1^Department of Physiology, Muhimbili University of Health and Allied Sciences, P.O. Box 65001, Dar es Salaam, Tanzania; ^2^Department of Epidemiology and Biostatistics, Muhimbili University of Health and Allied Sciences, P.O. Box 65001, Dar es Salaam, Tanzania; ^3^Management and Development for Health, Mwai Kibaki Road, Mikocheni, P.O. Box 79810, Dar es Salaam, Tanzania; ^4^Department of Behavioral Sciences, Muhimbili University of Health and Allied Sciences, P.O. Box 65001, Dar es Salaam, Tanzania

## Abstract

**Background:**

HIV infection and long-term HAART use are associated with metabolic and morphological changes. We assessed prevalence, types, and risk factors associated with lipodystrophy among HIV-infected adults attending CTC in Dar es Salaam, Tanzania.

**Methods:**

Analysis included 466 HIV-infected patients. Study protocol involved administration of structured questionnaire to collect sociodemographic and clinical information. Diagnosis of lipodystrophy was based on physician clinical assessment.

**Results:**

Lipodystrophy was present in 95 (20.4%) of the study participants, with lipoatrophy being the most common (49.5%) followed by mixed lipodystrophy (37.9%), and lipohypertrophy was the least prevalent (12.6%). Male gender, older age, long duration on HAART, and use of Stavudine containing regimen were associated with lipodystrophy (all *p* < 0.05). The risk for lipodystrophy was 1.6 times (AOR = 1.66, 95% CI = 1.01–2.72) for male participants and 13.3 times (AOR = 13.3, 95% CI = 6.4–27.7) for those on HAART. Long duration on HAART and use of Stavudine containing regimen were also associated with increased risk for lipodystrophy. Lipodystrophy was associated with poor perception about own body image and decreased social interactions.

**Conclusions:**

Lipodystrophy is common among HIV-infected patients in Tanzania, especially among male patients and those on HAART. Regular screening, monitoring, and patient awareness are needed for early identification and appropriate management.

## 1. Introduction

The recent estimates by UNAIDS indicate that 36.7 million [34.0 million–39.8 million] people were living with HIV in 2015 with Sub-Saharan Africa (SSA) bearing an inordinate share (70%) of the global HIV burden [1]. The number of HIV-infected people accessing Highly Active Antiretroviral Therapy (HAART) increased from 5.2 million in 2009 to 17.0 million in 2015 [1]. Of the estimated 1.1 million AIDS-related deaths in 2015, nearly three-quarter (73%) occurred in SSA [1]. In Tanzania, HIV incidence slowed to about 3.4 per 1000 person-years between 2004 and 2008 [2]. Data from the 2012 Tanzania HIV/AIDS and Malaria Indicator Survey (THMIS) indicated the national prevalence of HIV was 5.1% among the sexually active populations (15–49 years) and was higher among women (6.2%) compared to 3.8% in men [3].

The introduction of HAART provided HIV/AIDS patients with opportunity for quality of life improvement by inhibiting disease progression and development of opportunistic infections [4, 5]. Studies have indicated decreased HIV transmission and AIDS-related morbidity and mortality in both developed and developing countries [6, 7] resulting from high coverage of HAART. The provision of HIV services including HAART in Tanzania began in October 2004 with triple therapy consisting of 2 Nucleoside Reverse Transcriptase Inhibitors (NRTIs) + 1 Non-Nucleoside Reverse Transcriptase Inhibitor (NNRTI), 2 NRTIs + 1 Protease Inhibitor (PI), or 3 NRTIs being recommended [8]. The current National Guidelines for Management of HIV and AIDS provides drug combinations options for first-line treatment according to indications and contraindications. However, the current default first-line regimen in Tanzania is Tenofovir (TDF) 300 mg/Lamivudine (3TC) 300 mg/Efavirenz (EFV) 600 mg once daily at night [9]. Introduction of fixed dose combination therapy has significantly reduced pill burden to HIV patients and improved adherence to treatment [10, 11].

HIV infection and HAART have been associated with development of cardiovascular and metabolic complications including lipodystrophy [12, 13]. These effects are mediated through mitochondrial toxicity [13], atherosclerosis, and increased levels of inflammatory markers such as C-reactive protein, interleukin 6, cystatin C, and D-dimer all of which are associated with CVD risk [14, 15]. Lipodystrophy syndrome encompasses three clinical conditions characterized by abnormal body fat distribution; (i) lipoatrophy (peripheral fat wasting), (ii) lipohypertrophy (central fat accumulation), and (iii) mixed lipodystrophy (combination of both lipoatrophy and lipohypertrophy). Lipodystrophy patients have characteristic peripheral fat loss in the face, limbs, and buttocks, accompanied by central fat accumulation in the abdomen and breasts and over the dorsocervical spine (“buffalo hump”) and lipomas [16]. Protease Inhibitors (PI) containing regimen have been most strongly linked to body fat maldistribution syndrome [17, 18]. Non-Nucleoside Reverse Transcriptase Inhibitors (NRTIs) especially Stavudine have also been associated with body fat maldistribution [19]. The overall prevalence of at least one physical abnormality related to body fat maldistribution has been estimated at about 50% after one year of using HAART [20]. Other factors, such as duration of HIV infection, age, and gender, may also contribute to the risk of development of lipodystrophy [21]. Studies have shown that body fat changes are associated with psychological trauma which may be severe enough to affect a patient's desire to continue with HAART, limit therapy options, and profoundly affect the quality of life [22].

As Tanzania scales up HAART to all HIV-infected individuals country-wide through the “test and treat” strategy, it is critical to understand the proportion of HIV-related body fat maldistribution among HIV-infected people on treatment, so as to design and institute interventions that will minimize the risk for lipodystrophy. This study was therefore conducted to determine the prevalence, types, and risk factors associated with lipodystrophy among HIV-infected adults attending Care and Treatment Clinic (CTC) at three municipal referral hospitals in Dar es Salaam, Tanzania.

## 2. Materials and Methods

### 2.1. Study Design, Site, and Participants

This cross-sectional analytical study was conducted at Amana, Temeke, and Mwananyamala Municipal Hospitals in Dar es Salaam. At the time of data collection, Amana Hospital CTC had 14,133 active clients (with 7,932 on treatment and 6,201 under care and monitoring); Temeke Hospital CTC clinic had 16,530 active clients (with 8,871 on treatment and 7,659 under care and monitoring), and Mwananyamala Hospital CTC had 17,746 active clients (with 9,193 on treatment and 8,553 under care and monitoring).

Dar es Salaam region has a population of more than 4.3 million people, with an HIV prevalence of 5.1% among sexually active age group [3]. Study participants were HIV-positive patients aged ≥ 18 years who had been attending CTC at the three aforementioned municipal referral hospitals for at least 24 months.

### 2.2. Eligibility and Sampling Procedure

Stratified sampling was used to enroll participants into the study. The daily appointment list of patients was obtained from the hospital CTC records one day in advance. From this list the two groups (HAART and HAART naïve) and duration for which the patient has been attending CTC were determined. The list of patients who had attended CTC clinic for ≥24 months formed the sampling frame. Selection of patients from each stratum was done using simple random sampling techniques. Inclusion criteria were men and women aged ≥ 18 years, had attended CTC for ≥24 months, on HAART or HAART naïve, were clinically stable with good general condition, and provided a signed informed consent to participate. Pregnant women and very sick patients (Karnofsky score ≥ 40%) [23] were excluded from the study.

### 2.3. Recruitment of Research Assistants and Training

Six research assistants (3 physicians and 3 nurses) working at the CTC in the three municipal hospitals were recruited to assist during data collection. Selected study physicians and nurses had experience working at the CTC and had good interview and communication skills. Study physicians and nurses received training on the objectives of the study, recruitment, and assessments procedures, as well as importance of ensuring data quality.

### 2.4. Study Protocol

The study protocol involved administration of a questionnaire and physical examination. Sociodemographic information including age, sex, marital status, level of education, source of income, and lifestyle related information (smoking, alcohol drinking, type of diet in the last 24 hours, and physical activity) was assessed. Dietary assessment was conducted using a 24 h recall system. Based on a list of common foods, which was provided to them at the time of interview, subjects were also asked about food and/or beverages in the past 24 hours. A quick assessment of level of physical activity was conducted using the same interview guide. Participants were then asked to rank themselves in terms of level of physical activity. Duration and type of HAART medications and for HAART naïve the duration since HIV was diagnosed were ascertained from the patient medical records.

### 2.5. Anthropometric Measurement

Anthropometric measurements including height, weight, and waist and hip circumference were obtained following standardized procedures. Body Mass Index (BMI) was calculated as weight in kilograms divided by height in meters squared (kg/m^2^), and categorized as underweight (BMI ≤ 18.5 kg/m^2^), normal weight (BMI: 18.5–24.9 kg/m^2^), overweight (BMI: 25.0–29.9 kg/m^2^), and obese (BMI ≥ 30 kg/m^2^). Central obesity was defined as waist circumference ≥ 102 centimeters in men and ≥88 centimeters in women [24].

### 2.6. Assessment and Definition of Lipodystrophy Syndrome

In order to characterize self-rated body fat maldistribution, study participants were asked if they had noticed any changes that had alerted them in the following:

- Increased fat under the chin
- Increased fat on the back of the neck
- Increased abdominal girth
- Increased chest or breast fat
- Loss of fat in the face
- Loss of fat in the arms
- Loss of fat in the buttocks
- Loss of fat in the legs.

Information reported by the patient was validated by the physician after conducting a thorough physical examination. Fat maldistribution was scored as none (score = 0), mild (score = 1), moderate (score = 2), and severe (score = 3) and was recorded separately for fat losses and accumulations [25]. Lipodystrophy was categorized as

- *lipoatrophy*: presence of at least one site with moderate or severe fat loss,
- *lipohypertrophy*: presence of at least one site with moderate or severe fat accumulation, excluding isolated abdominal fat accumulation,
- *mixed lipodystrophy*: presence of signs of both lipoatrophy and lipohypertrophy.

### 2.7. Ethical Clearance

Ethical approval to conduct the study was obtained from Research Ethics Committee of the Muhimbili University of Health and Allied Sciences. Permission to conduct the study at the municipal hospitals was obtained from respective municipal medical officers of health through municipal research coordinators, who notified the medical officers in charge of the municipal hospitals. Medical officers in charge are overseers of these hospitals and facilitated to ensure that data collection was conducted smoothly.

The purpose of the study, expected duration of the participant's involvement, and description of the study procedures to be followed were clearly explained to potential participants. Description of any reasonably foreseeable benefits to the participant, such as awareness of body fat maldistribution risk factors and how they are going to be protected, for example, treatment of hypercholesterolemia, proper diet, and adequate physical activity, was done before enrolment. Risks or discomforts such as longer duration of interviews were also communicated in advance. Only those who agreed by signing informed consent were interviewed and were free to withdraw from the study during interview. To ensure confidentiality, participants were assigned unique identification numbers that were used during data collection.

### 2.8. Data Collection and Management

Data collected was edited during and after collection and adjusted for some missing information. The principal investigator scheduled visits to the study sites to observe and supervise data collection as per interview guide to ensure quality data. The principal investigator was also available for clarifications as another means of quality check. Therefore, the risk of study physicians and nurses recording wrong responses on account of misunderstanding the questions was minimized. Coding for open ended questions was done by the team and thereafter data was entered by the trained data entrant. Data cleaning and analysis were done using SPSS version 20.0 for windows.

### 2.9. Data Analysis

Frequency distribution was done to all variables to assess their distribution in the study population. Two-way tables were used to assess the association between dependent and independent variables by using the *χ*^2^-test. Multiple logistic regressions were used to assess individual contribution of each independent variable in predicting body fat maldistribution while controlling for confounding variables. In all the analyses, the *p* values were 2-sided and *p* ≤ 0.05 was considered statistically significant.

## 3. Results

### 3.1. Sociodemographic and General Characteristics of Study Sample

A total of 466 HIV-infected patients attending CTC at Amana, Temeke, and Mwananyamala Municipal Hospitals were included in the analysis. Demographic and general characteristics of the study participants are summarized in Table 1. Three-quarter (74.9%) of the participants were females. The mean age of the study participants was 41.1 ± 9.8 years, with majority (41.6%) being in the age group of 31–50 years. Nearly half (47.0%) of the participants were married or cohabiting, and majority had primary level of education (76.4%). Half of the participants (51.1%) were on HAART, with 49.6% of them being on Efavirenz based regimen, 36.1% on Stavudine based regimen, 13.0% on Nevirapine based regimen, and only 1.3% on Protease Inhibitor based second-line regimen. Majority (62.6%) of HAART patients were on treatment for 24–48 months.

### 3.2. Prevalence of Lipodystrophy

Prevalence of lipodystrophy was 20.4% with primary lipoatrophy (49.5%) being the most prevalent form of lipodystrophy followed by mixed lipodystrophy (37.9%) and primary lipohypertrophy (12.6%) was the least common (Table 1). A similar trend was observed for self-rating where 114 (24.5%) reported to have noted changes in body fat maldistribution, with more participants reporting decreased size of their body parts (17.5%) compared to increased size of body parts (14.0%).

### 3.3. Factors Associated with Lipodystrophy

Table 2 presents the characteristics of HIV-infected patients with lipodystrophy. Male participants were affected more than female participants (26.5% versus 18.3%; *p* = 0.058). However, the overall prevalence of overweight and that of obesity was significantly higher among women compared to men (25.8% versus 17.2%) for overweight and (16.3% versus 4.3%) for obesity (results not shown). Participants in the age group 41–50 years had the highest proportion of participants with lipodystrophy (27.6%) followed by those aged > 50 years (25.4%) and low proportion for younger participants (*p* = 0.003).

As for HAART status, lipodystrophy was higher (36.1% versus 3.9%) among participants on HAART compared to HAART naïve participants (*p* < 0.001). Differences in prevalence of lipodystrophy were also observed with the type of HAART used. Prevalence of lipodystrophy was highest for participants receiving second-line PI based regimen (66.7%) followed by Stavudine based regimen (53.5%). Nevirapine and Efavirenz based regimens had relatively similar rates of lipodystrophy (25.8% and 25.4%, resp.). The differences in the prevalence of lipodystrophy by type of HAART were statistically significant (*p* < 0.001). Duration of HAART use showed a U-shaped relationship with lipodystrophy. Patients on HAART for >48 months had highest proportion of lipodystrophy followed by those on HAART for 24 months and low in patients on HAART for 25–48 months (*p* < 0.001)

Lipodystrophy was highest among underweight patients (27.6%) followed by normal weight patients (21.7%) and least among obese patients (8.1%). The difference was statistically significant (*p* = 0.048). Although it did not attain statistical significance, prevalence of lipodystrophy was slightly higher among patients with central obesity (22.0%) compared to those without central obesity (19.6%).

### 3.4. Factors Associated with Different Types of Lipodystrophy

Table 3 presents analysis of factors associated with different types of lipodystrophy. Prevalence of primary lipoatrophy was significantly higher for patients aged more than 40 years (*p* = 0.004), male gender (*p* = 0.004), HAART use, longer duration of >48 months, and Stavudine based regimen (all *p* < 0.01). As for primary lipohypertrophy, significant association was observed with only duration of HAART use (*p* = 0.025) and was highest for patients on HAART for 24 months. Mixed type lipodystrophy was found to be associated with education level (*p* = 0.039), HAART use (*p* < 0.001), Protease Inhibitor based regimen (*p* = 0.013), and using HAART for 24–48 months.

### 3.5. Determinants of Lipodystrophy

Table 4 shows the results of multiple logistic regressions to determine the most important contributors to lipodystrophy in this population. A model for this analysis contained covariates that attained a statistically significant difference of *p* < 0.05 during bivariate analysis. These included age, gender, duration, and types of HAART used. While being in the age group of 31–40 years showed a protective effect (AOR = 0.18, 95% CI = 0.05–0.65), older age > 50 years showed a nonsignificant 12% increased risk for lipodystrophy (AOR = 1.12, 95% CI 0.58–2.19). Male gender was associated with 66% (AOR = 1.66, 95% CI = 1.01–2.72) increased risk for lipodystrophy compared to female gender. Using HAART was associated with 13 times increased risk for lipodystrophy. Similarly, long duration of Stavudine based regimen was significantly associated with 3 times increased risk for lipodystrophy (AOR = 3.22, 95% CI = 1.17–8.81).

### 3.6. Perceptions Related to Body Image and Quality of Life

Participants' perceptions about their body image and social life are summarized in Table 5. About 15.8% of the participants felt strongly that their current body outlook/image was worse compared to the way they looked before and 10.5% reported to dislike their mirror images. On average 24.5% of the participants with lipodystrophy avoided wearing clothing that display their body and 7.4% did so much more often. As for social aspects of life, 11.6% of the participants worried much about going into gatherings. Others reported on average lack of desire for sexual intercourse (9.7%), being afraid to be introduced to new people (13.7%), and avoiding outdoor activities like swimming (16.0%).

## 4. Discussion

This study aimed at determining the prevalence and factors associated with lipodystrophy, and assessing the perceptions towards body fat maldistribution among HIV-infected individuals attending CTC at three municipal hospitals in Dar es Salaam. Since the advent of HAART globally in 1996, HIV-related morbidity and mortality have decreased significantly. Now patients are living longer but chronic health complications such as cardiovascular diseases and lipodystrophy represent important health issues in this patient population.

In this analysis, the prevalence of physician diagnosed lipodystrophy was 20.4%. The combined prevalence of overweight and obesity was 37.0%, whereas that of central obesity was 35.7%. The precise definition and estimation of lipodystrophy are important for comparison across studies and populations. Prevalence observed in this study was low compared to findings from studies conducted in Western populations [26, 27]. Conversely, it was considerably higher than 6.6% reported among African patients after 18 months of HAART [28]. Two other studies conducted in Africa demonstrated higher prevalence rates of lipodystrophy compared to our study [29, 30]. The observed low prevalence of lipodystrophy in comparison to objective measures (BMI and waist circumference) and that of other studies conducted in Africa may be caused by clinical diagnosis by the attending physician which is subjective and compounded by malnutrition seen in HIV-positive.

Our study has demonstrated an association between lipodystrophy and demographic characteristics including age, gender, and level of education. In our study the prevalence of lipodystrophy was higher in men than women. Conversely, the prevalence of overweight and obesity as defined by BMI was higher in women compared to men. As for age, lipodystrophy was found to be higher for participants aged 41 years and above. Findings from Rwandan study indicated that age and gender were significantly associated with lipodystrophy [29]. Several other studies have also found a similar association [31, 32]. In African settings, more women than men are generally obese [33] and obesity tends to increase with age and level of education [34].

Type, duration, and current use of HAART especially PI and NNRTI containing regimen are strongly associated with development of severe lipoatrophy [35]. In our study, lipodystrophy was highest for patients on PI based second-line HAART regimen, followed by patients on Stavudine based regimen and Nevirapine based regimen, and least among patients on Efavirenz based regimen. Prospective studies investigating body composition in patients starting HAART for the first time [35, 36] have demonstrated initial increases in limb fat during the first few months of therapy, followed by progressive decline during ensuing three years. In one study the decline was estimated to be 14% per year among white men receiving regimens containing Stavudine or Zidovudine with Lamivudine and either a Protease Inhibitor or Non-Nucleoside Reverse Transcriptase Inhibitor [36].

Our findings on association of Stavudine and Efavirenz with lipodystrophy are consistent with other studies that used Stavudine and Efavirenz based therapy [29, 37]. Non-Nucleoside Reverse Transcriptase Inhibitors (NNRTIs), such as Efavirenz, have been associated with in vitro altered deposition of triglycerides in the adipocyte; however its clear clinical implications is still being studied. Thymidine analog Nucleoside Reverse Transcriptase Inhibitors (tNRTIs) are risk factors for mitochondrial dysfunction associated with both dyslipidemia and lipoatrophy (particularly in subcutaneous adipocytes) [37]. Changing patients from thymidine analogs to NRTIs has demonstrated beneficial effects on lipoatrophy [38]. Stavudine, the nucleoside analog with the highest propensity for causing mitochondrial dysfunction, is relatively inexpensive and widely available in the developing world. Zidovudine is also relatively available and has many therapeutic advantages but also carries risk of producing mitochondrial toxicity. Hence the use of these drugs in the developing world will lead to mitochondrial toxicity and development of lipodystrophy as is shown in this study.

This study found diverse emotional feelings among participants regarding perception about their body image and quality of life, ranging from feeling worse than before and feeling bad about own mirror image to avoiding wearing some dressing because of fear of stigma. However, we did not explore the desire to stop treatment because of the change in body image. Other studies have also explored the psychosocial effects of lipodystrophy among HIV patients on HAART [39, 40]. Lipodystrophy might have substantial psychological repercussions with subsequent negative impact on the patient's quality of life, emergence of depressive symptoms, or anxiety [41, 42]. Moreover, body change status and subsequent stigmatization could produce social isolation and distress and, even, change in beliefs about drugs and consequently decreased adherence to HAART.

As with regard to social life, participants with lipodystrophy reported isolating themselves from other people, tending to avoid outside activities like swimming, avoiding events that will have many people, and a diminished desire for sexual intercourse. Other studies have also demonstrated altered quality of life among patients with lipodystrophy [43]. Body-shape changes may affect patients' psychosocial function and quality of life and may lead to patients considering cessation of treatment [44]. However, few studies to date have formally addressed the impact of these changes on the patient. Hence this study demonstrates that there are significant variations in perceptions about body changes and the quality of life of these patients that need to be addressed in a larger and well-designed study for that purpose.

There are few limitations to our analysis. The cross-sectional design does not provide causal association of studies parameters with lipodystrophy. The study included patients who were attending CTC clinics and may not be representative of all HIV-infected individuals. However, the fact that the study was conducted in Dar es Salaam which is metropolitan with a diverse population from nearly all other regions makes it possible for the findings to be generalizable to the broader Tanzanian population. We did not collect detailed information on patient's treatment history. For participants who changed treatment due to side effects, the observed association of HAART regimen and lipodystrophy could have been caused by the previous regimen. Therefore we may have missed the true association of HAART with lipodystrophy. The detailed treatment history could also provide some insights on the quality of care in terms of management for lipodystrophy through change of regimen implicated in causing lipodystrophy. Assessments of body fat changes were done clinically, instead of using specialized instruments such as dual energy X-ray absorptiometry (DEXA) or linear calipers. Nevertheless, subjective assessment is strongly correlated with DEXA [45].

In conclusion, prevalence of lipodystrophy among HIV-infected patients is high and is worsened by HAART use. Our findings clearly demonstrate the long-term side effects of HAART, particularly Stavudine based regimen among HIV-infected patients in Tanzania. Treatment modalities among patients with lipodystrophy was not a scope of this study; however if these patients were adequately treated their perceptions against lipodystrophy could improve their quality of life. Deliberately sought efforts are needed to incorporate management of lipodystrophy and other long-term complications of HAART in Tanzania in the era of test and treat for improved adherence and overall improved patient care.

## Figures and Tables

**Table 1:** Sociodemographic and general characteristics of the study participants.

Characteristic	Number (%)
Age (years)	
≤30 years	51 (10.9)
31–40 years	194 (41.6)
41–50 years	134 (28.8)
≥51 years	67 (14.4)
Missing	20 (4.3)
Gender	
Male	117 (25.1)
Female	349 (74.9)
Marital status	
Married	219 (47.0)
Single	89 (19.1)
Divorced/separated	81 (17.4)
Widowed	77 (16.5)
Education level	
No formal education	27 (5.8)
Primary education	356 (76.4)
Secondary education	68 (14.6)
College/university	15 (3.2)
CTC of enrolment	
Amana Hospital	152 (32.6)
Temeke Hospital	155 (33.3)
Mwananyamala Hospital	159 (34.1)
HAART use	
On HAART	238 (51.1)
HAART naïve	228 (48.9)
Type of HAART	
Nevirapine based regimen	31 (13.0)
Efavirenz based regimen	118 (49.6)
Stavudine based regimen	86 (36.1)
Protease Inhibitor based regimen	3 (1.3)
Duration of HAART use	
HAART naïve	228 (48.9)
24 months	60 (12.9)
25–48 months	149 (32.0)
>48 months	29 (6.2)
Lipodystrophy	
Yes	95 (20.4)
No	371 (79.6)
Types of lipodystrophy	
Primary lipoatrophy	47 (49.5)
Primary lipohypertrophy	12 (12.6)
Mixed lipodystrophy	36 (37.9)
Body Mass Index (BMI)	
Underweight	58 (12.5)
Normal weight	235 (50.5)
Overweight	110 (23.7)
Obese	62 (13.3)
Central obesity	
No	301 (64.7)
Yes	164 (35.7)

BMI: Body Mass Index; CTC: Care and Treatment Clinic; HAART: Highly Active Antiretroviral Therapy.

**Table 2:** Factors associated with lipodystrophy among HIV-infected patients in Dar es Salaam.

Characteristic	Lipodystrophy		*p* value
	Present	Absent	
Age (years)			
≤30	3 (5.9)	48 (94.1)	**0.003**
31–40	32 (16.5)	162 (83.5)	
41–50	37 (27.6)	97 (72.4)	
>50	17 (25.4)	50 (74.6)	
Missing	6 (30.0)	14 (70.0)	
Gender			
Male	31 (26.5)	86 (73.5)	0.058
Female	64 (18.3)	285 (81.7)	
Marital status			
Married	41 (18.7)	178 (81.3)	0.860
Single	20 (22.5)	69 (77.5)	
Divorced/separated	17 (21.0)	64 (79.0)	
Widowed	17 (22.1)	60 (77.9)	
Education level			
No formal education	6 (22.2)	21 (77.8)	0.484
Primary education	68 (19.1)	288 (80.9)	
Secondary education	16 (23.5)	52 (76.5)	
College/university	5 (33.3)	10 (66.7)	
HAAT use			
On HAART	86 (36.1)	152 (63.9)	**<0.001**
HAART naïve	9 (3.9)	219 (96.1)	
Type of HAART			
Nevirapine based regimen	8 (25.8)	23 (74.2)	**<0.001**
Efavirenz based regimen	30 (25.4)	88 (74.6)	
Stavudine based regimen	46 (53.5)	40 (46.5)	
Protease Inhibitor based regimen	2 (66.7)	1 (33.3)	
Duration of HAART use			
HAART naïve	9 (3.9)	219 (96.1)	**<0.001**
24 months	23 (38.3)	37 (61.7)	
24–48 months	51 (34.2)	98 (65.8)	
>48 months	12 (41.4)	17 (58.6)	
Body Mass Index (BMI)			
Underweight	16 (27.6)	42 (72.4)	**0.048**
Normal weight	51 (21.7)	184 (78.3)	
Overweight	23 (20.9)	87 (79.1)	
Obese	5 (8.1)	57 (91.9)	
Central obesity			
No	59 (19.6)	242 (80.4)	0.630
Yes	36 (22.0)	128 (78.0)	

BMI: Body Mass Index; HAART: Highly Active Antiretroviral Therapy.

**Table 3:** Factors associated with different types of lipodystrophy among HIV-infected patients in Dar es Salaam.

Characteristic	Lipoatrophy			Lipohypertrophy			Mixed lipodystrophy		
	Yes	No	*p* value	Yes	No	*p* value	Yes	No	*p* value
Age categories (yrs)									
≤30	0 (0.0)	51 (100.0)	**0.004**	1 (2.0)	50 (98.0)	0.463	2 (3.9)	49 (96.1)	0.436
31–40	13 (6.7)	181 (93.3)		6 (3.1)	188 (96.9)		13 (6.7)	181 (93.3)	
41–50	18 (13.4)	116 (86.6)		5 (3.9)	129 (96.3)		14 (10.4)	120 (89.6)	
≥51	11 (16.4)	56 (83.6)		0 (0.0)	67 (100.0)		6 (9.0)	61 (91.0)	
Missing	5 (33.3)	15 (66.7)		0 (0.0)	20 (100.0)		2 (9.5)	19 (90.5)	
Gender									
Male	20 (17.1)	97 (82.9)	**0.004**	3 (2.6)	114 (97.4)	1.000	8 (6.8)	109 (93.2)	0.699
Female	27 (7.7)	322 (92.3)		9 (2.6)	340 (97.4)		28 (8.0)	321 (92.0)	
Education level									
No formal education	3 (11.1)	24 (88.9)	0.797	1 (3.7)	26 (96.3)	0.204	2 (7.4)	25 (92.6)	**0.039**
Primary education	34 (9.6)	322 (90.4)		7 (2.0)	349 (98.0)		27 (7.6)	329 (92.4)	
Secondary education	9 (13.2)	59 (86.8)		4 (5.9)	64 (94.1)		3 (4.4)	65 (95.6)	
College/university	1 (6.7)	14 (93.3)		0 (0.0)	15 (100.0)		4 (26.7)	11 (73.3)	
HAART use									
On HAART	42 (17.6)	196 (82.4)	**<0.001**	9 (3.8)	229 (96.2)	0.142	35 (14.7)	203 (85.3)	**<0.001**
HAART naïve	5 (2.2)	223 (97.8)		3 (1.3)	225 (98.7)		1 (0.4)	227 (99.6)	
Type of HAART used									
Nevirapine based	1 (3.2)	30 (96.8)	**0.002**	2 (6.5)	29 (93.5)	0.817	5 (16.1)	26 (83.9)	**0.013**
Efavirenz based	15 (12.7)	103 (87.3)		4 (3.4)	114 (96.6)		11 (9.3)	107 (90.7)	
Stavudine based	26 (30.2)	60 (69.8)		3 (3.5)	83 (96.5)		17 (19.8)	69 (80.2)	
Protease Inhibitor based	0 (0.0)	3 (100.0)		0 (0.0)	3 (100.0)		2 (66.7)	1 (33.3)	
Duration of HAART use									
HAART naïve	5 (2.2)	223 (97.8)	**<0.001**	3 (1.3)	225 (98.7)	**0.025**	1 (0.4)	227 (99.6)	**<0.001**
24 months	11 (18.3)	49 (81.7)		5 (8.3)	55 (91.7)		7 (11.7)	53 (88.3)	
24–48 months	22 (14.8)	127 (85.2)		3 (2.0)	146 (98.0)		26 (17.4)	123 (82.6)	
>48 months	9 (31.0)	20 (69.0)		1 (3.4)	28 (96.6)		2 (6.9)	27 (93.1)	

HAART: Highly Active Antiretroviral Therapy.

**Table 4:** Determinants of lipodystrophy among HIV-infected patients in Dar es Salaam.

Variables	AOR	95% CI	*p* value
Age (years)			
≤30	Ref		
31–40	0.18	0.05–0.65	**0.009**
41–50	0.59	0.30–1.15	0.120
≥51	1.12	0.58–2.19	0.735
Gender			
Female	Ref		
Male	1.66	1.01–2.72	**0.045**
HAAT use			
HAART naïve	Ref		
On HAART	13.3	6.4–27.7	**<0.001**
Type of HAART used			
Nevirapine based	Ref		
Efavirenz based	0.98	0.36–2.66	0.962
Stavudine based	3.22	1.17–8.81	**0.023**
Protease Inhibitor based	1.06	0.08–9.02	1.00
Duration of HAART use			
HAART naïve	Ref		
24 months	12.8	6.0–27.6	**<0.001**
25–48 months	13.6	5.7–32.4	**<0.001**
>48 months	15.3	5.3–43.8	**<0.001**

AOR: Adjusted Odds Ratio; CI: Confidence Interval; HAART: Highly Active Antiretroviral Therapy.

**Table 5:** Changes in body parts and perceptions about current body image among HIV infected patients in Dar es Salaam.

Change and perception	Extent of perceived change			
	None	Very little	Average	Very much
Increased body parts				
Neck and chin	88 (93.6)	2 (2.1)	4 (4.3)	0 (0.0)
Back of shoulders	83 (89.2)	2 (2.2)	8 (8.6)	0 (0.0)
Breast and/or chest	71 (76.3)	7 (7.5)	14 (15.1)	1 (1.1)
Abdomen	66 (69.5)	7 (7.4)	21 (22.1)	1 (1.1)
Waist	78 (85.7)	2 (2.2)	10 (11.0)	1 (1.1)
Decreased body parts				
Face	53 (57.0)	9 (9.7)	25 (26.9)	6 (6.5)
Upper limbs	49 (53.8)	15 (16.5)	19 (20.9)	8 (8.8)
Lower limbs	49 (53.3)	18 (19.6)	17 (18.5)	8 (8.7)
Buttocks	54 (57.4)	9 (9.6)	24 (25.5)	7 (7.4)
Perception about body image				
Angry about own body	60 (63.8)	12 (12.8)	18 (19.1)	4 (4.3)
Avoiding some clothing	50 (53.2)	14 (14.9)	23 (24.5)	7 (7.4)
Ashamed of own body	63 (66.3)	9 (9.5)	19 (20.0)	4 (4.2)
Unhappy about mirror image	49 (51.6)	16 (16.8)	20 (21.1)	10 (10.5)
Conscious of other people	69 (75.8)	16 (17.6)	5 (5.5)	1 (1.1)
Bad body shape than before	50 (52.6)	10 (10.5)	20 (21.1)	15 (15.8)
Afraid to mix with other people	76 (80.0)	11 (11.6)	8 (8.4)	0 (0.0)
Perceptions related to social life				
Avoiding outdoor activities	67 (71.3)	10 (10.6)	15 (16.0)	2 (2.1)
Avoiding social events with many people	76 (78.4)	7 (7.2)	12 (12.4)	2 (2.1)
Lack of desire for sexual intercourse	73 (78.5)	8 (8.6)	9 (9.7)	3 (3.2)
Afraid to be introduced to new people	73 (76.8)	9 (9.5)	13 (13.7)	0 (0.0)
Afraid to enter a room with new people	79 (83.2)	7 (7.4)	9 (9.5)	0 (0.0)
Habit to exclude himself from other people	82 (88.2)	6 (6.5)	4 (4.3)	1 (1.1)
Avoid going into social gatherings/parties	61 (64.2)	7 (7.4)	16 (16.8)	11 (11.6)
